# Relative Effectiveness of the MF59®‐Adjuvanted Influenza Vaccine Versus High‐Dose and Non‐Adjuvanted Influenza Vaccines in Preventing Cardiorespiratory Hospitalizations During the 2019–2020 US Influenza Season

**DOI:** 10.1111/irv.13288

**Published:** 2024-04-21

**Authors:** Mahrukh Imran, Joan Puig‐Barbera, Justin R. Ortiz, Lorena Lopez‐Gonzalez, Alex Dean, Machaon Bonafede, Mendel D. M. Haag

**Affiliations:** ^1^ CSL Seqirus Inc. Kirkland Quebec Canada; ^2^ FISABIO Valencia Spain; ^3^ University of Maryland School of Medicine Baltimore Maryland USA; ^4^ Veradigm Chicago Illinois USA; ^5^ CSL Seqirus Netherlands B. V Amsterdam The Netherlands

**Keywords:** adjuvanted influenza vaccine, high‐dose influenza vaccine, hospitalizations, influenza, ischemic stroke, myocardial infarction, older adults, pneumonia, quadrivalent inactivated influenza vaccine, relative vaccine effectiveness

## Abstract

**Background:**

Adults ≥ 65 years of age have suboptimal influenza vaccination responses compared to younger adults due to age‐related immunosenescence. Two vaccines were specifically developed to enhance protection: MF59‐adjuvanted trivalent influenza vaccine (aIIV3) and high‐dose egg‐based trivalent influenza vaccine (HD‐IIV3e).

**Methods:**

In a retrospective cohort study conducted using US electronic medical records linked to claims data during the 2019–2020 influenza season, we compared the relative vaccine effectiveness (rVE) of aIIV3 with HD‐IIV3e and a standard‐dose non‐adjuvanted egg‐based quadrivalent inactivated influenza vaccine (IIV4e) for the prevention of cardiorespiratory hospitalizations, including influenza hospitalizations. We evaluated outcomes in the “any” diagnosis position and the “admitting” position on the claim. A doubly robust methodology using inverse probability of treatment weighting and logistic regression was used to adjust for covariate imbalance. rVE was calculated as 100 * (1 − OR_adjusted_).

**Results:**

The study included 4,299,594 adults ≥ 65 years of age who received aIIV3, HD‐IIV3e, or IIV4e. Overall, aIIV3 was associated with lower proportions of cardiorespiratory hospitalizations with diagnoses in any position compared to HD‐IIV3e (rVE = 3.9% [95% CI, 2.7–5.0]) or IIV4e (9.0% [95% CI, 7.7–10.4]). Specifically, aIIV3 was more effective compared with HD‐IIV3e and IIV4e in preventing influenza hospitalizations (HD‐IIV3e: 9.7% [95% CI, 1.9–17.0]; IIV4e: 25.3% [95% CI, 17.7–32.2]). Consistent trends were observed for admitting diagnoses.

**Conclusion:**

Relative to both HD‐IIV3e and IIV4e, aIIV3 provided improved protection from cardiorespiratory or influenza hospitalizations.

## 1 Introduction

Influenza virus infections are the ninth leading cause of death in the United States [1]. Severe complications of influenza include pneumonia and exacerbation of underlying ischemic heart disease and cerebrovascular disease [2, 3, 4]. The highest rates of influenza‐related deaths and hospitalizations occur among adults ≥ 65 years of age. For this reason, adults aged ≥ 65 years are considered a high‐priority vaccination group by the US Advisory Committee on Immunization Practices (ACIP) [5, 6].

Compared with immune responses in younger adults, older adults' immune systems tend to be less responsive to non‐adjuvanted standard‐dose (15 μg per antigen), egg‐grown, inactivated influenza vaccines [7, 8]. Approaches to overcome this age‐related immunosenescence include adding an adjuvant to the vaccine to enhance and broaden the recipient's immune response or increasing the dose of influenza virus antigens contained within the vaccine. Vaccines developed to fulfill these needs include an MF59®‐adjuvanted, egg‐grown trivalent inactivated influenza vaccine (aIIV3; Fluad®, Seqirus USA Inc., Summit, NJ, USA) with 15 μg per antigen and a high‐dose (60 μg per antigen), egg‐grown, trivalent inactivated influenza vaccine (HD‐IIV3e; Fluzone® High‐Dose, Sanofi Pasteur Inc., Swiftwater, PA, USA). The adjuvanted and high‐dose vaccines are licensed and available in the United States, the United Kingdom, Canada, Europe, Australia, and elsewhere worldwide.

In clinical studies, aIIV3 enhanced immune responses, including production of cross‐reactive antibodies, relative to standard influenza vaccines [9, 10, 11, 12]. Multiple studies have also shown that the effectiveness of aIIV3 matches or may even exceed that of non‐adjuvanted influenza vaccines—including HD‐IIV3e and IIV4e—in adults ≥ 65 years [7, 13, 14, 15, 16, 17, 18, 19, 20, 21, 22, 23]. In previous studies conducted during the 2017–2018, 2018–2019, and 2019–2020 influenza seasons in the United States, we assessed the relative vaccine effectiveness (rVE) of aIIV3 versus HD‐IIV3e and IIV4e for the prevention of influenza‐related medical encounters in adults ≥ 65 years of age [19]. Additional data on the effectiveness of aIIV3 in preventing cardiorespiratory hospitalizations are important to understand the broader public health benefit of aIIV3. In this study, we compared aIIV3 to HD‐IIV3e and IIV4e for the prevention of hospitalizations for cardiorespiratory conditions, including influenza hospitalizations, in adults ≥ 65 years of age during the 2019–2020 influenza season in the United States.

## 2 Methods

### 2.1 Study Design

We conducted a retrospective cohort study during the 2019–2020 US influenza season. The data comprised de‐identified medical records of US adults ≥ 65 years of age who received a seasonal influenza vaccination (aIIV3, HD‐IIV3e, or IIV4e) during the 2019–2020 influenza season. The study was designed, implemented, and reported in accordance with Good Pharmacoepidemiological Practice, applicable local regulations, and the ethical principles laid down in the Declaration of Helsinki and the Reporting of Studies Conducted using Observational Routinely Collected Health Data (RECORD) recommendations [24, 25].

### 2.2 Data Sources

For the analysis, we used an integrated dataset comprising US primary and specialty care data from Veradigm Health Insights electronic medical record (EMR) platforms (including components from Allscripts Tiers 1 and 2 [Chicago, IL] and Practice Fusion [San Francisco, CA]) linked with pharmacy and medical claims data from Komodo Health Inc. (New York, NY). The Veradigm EMR platform comprised all primary care interactions for over 120 million individuals at the time of study conduct. Komodo Health Inc. obtains data both directly from payers (closed claims) as well as from broad‐based healthcare sources, including clearinghouses, pharmacies, and software platforms, and can capture an individual's healthcare activities, regardless of their insurance provider (open/closed claims). This study used all available claims data for the analysis. The data sources have been described in more detail elsewhere [26].

An algorithm developed by Datavant (San Francisco, CA) was used to de‐identify individual‐specific information to certify privacy and meet minimum protected health information (PHI) standards in accordance with the Health Insurance Portability and Accountability Act (HIPAA) Privacy Rule. Individual‐level de‐identified tokens are generated deterministically in each data source, using fields such as name, date of birth, and gender. The final linked data set is created as a merge of the individual‐level de‐identified tokens in each individual dataset and contains no PHI. Research staff did not participate in the de‐identification process and had no access to the datasets until all identifying information had been removed. Because this study was a noninterventional, retrospective study using a certified HIPAA‐compliant database, institutional review board approval was not necessary.

### 2.3 Study Period

The observation period began August 1, 2019, and ended on March 7, 2020, to avoid outcome misclassification due to overlap with the severe acute respiratory syndrome coronavirus 2 (SARS‐CoV‐2) pandemic. To evaluate outcome specificity and assess the impact of SARS‐CoV‐2 circulation, we conducted sensitivity analyses using varying observation periods (defined in Section 2.8).

### 2.4 Study Population

The study population included individuals ≥ 65 years of age who resided in the United States and who received aIIV3, HD‐IIV3e, or IIV4e between August 1, 2019, and January 31, 2020 (vaccination identification period). Eligible subjects also had at least 1 year of primary care medical history in the Veradigm EMR dataset and recorded activity in the Komodo pharmacy or medical claims data prior to vaccination and after March 7, 2020. Subjects were excluded if they had a record of any type of influenza vaccination between May 19, 2019, and July 31, 2019; received > 1 influenza vaccine; had an influenza‐related medical encounter before becoming fully vaccinated (as elaborated below) or before September 29, 2019 (i.e., the start of the 2019–2020 influenza season as defined by the US Centers for Disease Control and Prevention [CDC] [27]); and or had missing age, sex, or geographic region data.

### 2.5 Influenza Vaccine Exposure

The vaccines of interest included aIIV3, HD‐IIV3e, or IIV4e given during the vaccination identification period. Codes for vaccines administered (CVX), Current Procedural Terminology (CPT) codes, and national drug codes (NDCs) were used to identify influenza vaccinated subjects from EMRs and claims data (Table S1). Subjects were considered fully vaccinated 14 days after vaccination to allow for development of vaccine‐specific antibodies over the following 2 weeks. Of note, quadrivalent formulations of aIIV3 and HD‐IIV3e are also available in the United States; however, they were not available for use during the study period.

### 2.6 Outcomes

Hospitalizations occurring at least 14 days after the influenza vaccination for cardiorespiratory conditions, including respiratory infections (i.e., influenza and pneumonia, evaluated separately), myocardial infarction (MI), and ischemic stroke, were included in the study if they occurred between September 30, 2019 (Week 40), and March 7, 2020 (Week 10). Outcomes were identified by relevant diagnosis codes in any position on the claim or restricted to only the admitting (also known as the primary) position on a claim (Table S2). Outcomes identified in the “admitting” diagnosis position are a subset of the “any” position; the “any” diagnosis category includes the admitting diagnosis as well as subsequent diagnosis regardless of the admitting diagnosis. Results for each type of claim are presented separately. Hospitalization for injury or trauma was evaluated as a negative control outcome.

### 2.7 Covariates

Covariates were ascertained from EMRs and claims in the 12 months prior to vaccination. These included age, sex, race, ethnicity, US geographic region, frailty index, week of vaccination (Table S3), body mass index (BMI < 30 or ≥ 30 kg/m^2^), smoking status, individual comorbidities included in the Charlson Comorbidity Index (CCI; Table S4) [28, 29], baseline cardiovascular risk (determined by history of hypercholesterolemia; hypertension; type 2 diabetes; obesity; and hospitalizations for MI, ischemic stroke, heart failure, and transient ischemic attack [TIA]), number of outpatient visits, and number of inpatient admissions.

### 2.8 Statistical Methodology

Standardized mean differences (SMD) were used to assess differences in baseline covariates between the exposure groups (aIIV3, HD‐IIV3e, and IIV4e); we chose a value of ≤ 0.1 to indicate a negligible difference. Inverse probability of treatment weighting (IPTW) was used to adjust for covariate imbalance between cohorts [30]. Propensity scores calculated for each exposure cohort using a multivariable logit model adjusted for all covariates listed above were used to create stabilized IPTWs, in which weights were truncated at the 99th percentile to attenuate any extreme variability from outlier subjects. Adjusted odds ratios (ORs) were estimated in the IPTW‐weighted sample using a multivariable logistic regression model that included all variables from the propensity score–generation model as covariates [31]. rVE was calculated as 100 * (1 − OR_adjusted_) and reported with 95% confidence intervals (CI). Missing demographic variables were reported as “missing” or “not reported.” Subjects with missing values for age or sex were excluded from the study. Analyses were conducted using SQL and SAS® (Version 9.4).

Two sensitivity analyses were used to evaluate the robustness of study assumptions. First, outcome measurement was restricted to the period of highest influenza activity as defined by the CDC using the moving epidemic method (MEM)—that is, December 8, 2019 (Week 50), through March 7, 2020 (Week 10) [32]. Second, to further mitigate the potential impact of SARS‐CoV‐2 cocirculation around the end of February and early March 2020 in the United States, another sensitivity analysis truncated the outcome ascertainment period to end on February 15, 2020 (Week 7).

### 2.9 Post Hoc Analyses

Emerging evidence demonstrated that older adults hospitalized for influenza may have an increased risk of injury or trauma hospitalizations [33]. Therefore, the following additional negative control outcomes were included as a post‐hoc analysis: appendicitis, cataracts, eyelid disorder, hemorrhoids, herpes zoster, lipoma, and nail disorders. Some of these negative control outcomes have been used in previous influenza vaccine effectiveness analyses [34].

## 3 Results

### 3.1 Study Subjects

The total study population comprised 4,299,594 subjects, of whom 1,083,466 (25.2%) received aIIV3; 2,448,403 (56.9%) received HD‐IIV3e; and 767,725 (17.9%) received IIV4e (Table 1). Subject characteristics on the vaccination date (i.e., index date) were mostly similar across vaccine groups (Table 2). After IPTW, cohorts were balanced, with SMDs < 0.1 observed for both comparison cohorts (Table 2).

**TABLE 1:** Subject selection criteria.

Selection criterion	*N* (%)
Subject had a vaccination with aIIV3, HD‐IIV3e, and IIV4e between August 1, 2019, and January 31, 2020 (vaccination identification period).	15,022,512 (100)
Subject had no evidence of vaccination for influenza between the end of the previous season's influenza season (May 19, 2019) and the start of the current season's vaccine identification period (July 31, 2019)	15,010,005 (99.9)
Subject had no evidence of > 1 influenza vaccine administration during the 2019–2020 influenza season.	14,136,465 (94.1)
Subject was ≥ 65 years old on the index date.	5,424,916 (36.1)
Subject had a transcript record in the Veradigm EMR ≥ 12 months before the vaccination date.	4,565,942 (30.4)
Subject had no evidence of an influenza‐related medical encounter prior to becoming fully vaccinated (i.e., vaccination date + 14 days) or prior the start of the influenza season (i.e., prior to September 30, 2019).	4,558,152 (30.3)
Subject did not have missing age, sex, or geographic region data	4,460,786 (29.7)
Subject had continuous enrollment with open/closed claims 6 months prior to index date and after study end.	4,299,594 (28.6)
Received aIIV3 influenza vaccine	1,083,466 (25.2)
Received HD‐IIV3e influenza vaccine	2,448,403 (56.9)
Received IIV4e influenza vaccine	767,725 (17.9)

Abbreviations: aIIV3, adjuvanted trivalent influenza vaccine; EMR, electronic medical record; HD‐IIV3e, high‐dose, egg‐based trivalent influenza vaccine; IIV4e, egg‐based quadrivalent influenza vaccine.

**TABLE 2:** Subject demographic and clinical characteristics at vaccination (index) date.^a^

	aIIV3 (*n* = 1,083,466)	HD‐IIV3e (*n* = 2,448,403)	IIV4e (*n* = 767,725)	Unweighted SMD		IPTW SMD	
				aIIV3 vs. HD‐IIV3e	aIIV3 vs. IIV4e	aIIV3 vs. HD‐IIV3e	aIIV3 vs. IIV4e
Mean age ± SD, years	74.8 ± 6.6	74.8 ± 6.7	74.0 ± 7.0	−0.01	0.14	0.0	0.0
Age group (*n*, %)							
65–74	577,392 (53.3)	1,306,094 (53.3)	446,563 (58.2)	0.00	−0.10	0.0	0.0
75–84	377,732 (34.9)	837,141 (34.2)	228,980 (29.8)	0.00	0.09	0.0	**0.1**
≥ 85	128,342 (11.8)	305,168 (12.5)	92,182 (12.0)	−0.01	0.02	0.0	0.0
Sex (*n*, %)							
Female	629,885 (58.1)	1,426,495 (58.3)	450,238 (58.6)	0.00	0.00	0.0	0.0
Male	453,581 (41.9)	1,021,908 (41.7)	317,487 (41.4)	0.00	0.00	0.0	0.0
Race (*n*, %)							
White	681,647 (62.9)	1,515,684 (61.9)	421,767 (54.9)	0.02	0.18	0.0	0.0
Black	41,626 (3.8)	113,429 (4.6)	43,675 (5.7)	−0.02	−0.07	0.0	0.0
Asian	16,251 (1.5)	51,302 (2.1)	34,592 (4.5)	−0.04	−0.15	0.0	**−0.1**
Other	23,973 (2.2)	58,103 (2.4)	25,399 (3.3)	−0.02	−0.08	0.0	0.0
Unknown/not reported	319,969 (29.5)	709,885 (29.0)	242,292 (31.6)	0.00	−0.08	0.0	0.0
Ethnicity (*n*, %)							
Hispanic	40,420 (3.7)	94,280 (3.9)	53,037 (6.9)	−0.01	−0.17	0.0	0.0
Non‐Hispanic	903,381 (83.4)	2,039,006 (83.3)	624,712 (81.4)	0.01	0.08	0.0	0.0
Unknown/not reported	139,665 (12.9)	315,117 (12.9)	89,976 (11.7)	0.00	0.04	0.0	0.0
Geographic region (*n*, %)							
Northeast	166,333 (15.4)	485,775 (19.8)	165,142 (21.5)	−0.08	−0.07	0.0	0.0
Midwest	176,400 (16.3)	542,493 (22.2)	143,588 (18.7)	−0.09	−0.02	0.0	0.0
South	614,061 (56.7)	929,901 (38.0)	276,404 (36.0)	**0.26**	**0.31**	**0.1**	**0.1**
West	126,672 (11.7)	490,234 (20.0)	182,591 (23.8)	**−0.16**	**−0.29**	**−0.1**	**−0.1**
Outpatient visits, mean ± SD	6.5 ± 7.0	6.8 ± 7.3	7.6 ± 7.8	−0.01	−0.05	0.0	0.0
Inpatient admissions, mean ± SD	0.2 ± 0.9	0.2 ± 0.9	0.3 ± 1.0	−0.01	−0.05	0.0	0.0
CCI score, mean ± SD	1.5 ± 1.8	1.6 ± 1.9	1.8 ± 2.0	−0.06	**−0.17**	0.0	0.0
CCI category, *n* (%)							
0	445,943 (41.2)	939,071 (38.4)	257,968 (33.6)	0.03	**0.12**	0.0	0.0
1	233,623 (21.6)	531,354 (21.7)	163,990 (21.4)	0.01	0.03	0.0	0.0
2	167,533 (15.5)	386,771 (15.8)	125,832 (16.4)	0.00	−0.01	0.0	0.0
3	102,375 (9.4)	243,100 (9.9)	85,604 (11.2)	−0.01	−0.04	0.0	0.0
≥ 4	133,992 (12.4)	348,107 (14.2)	134,331 (17.5)	−0.04	**−0.13**	0.0	0.0
Electronic frailty index score, mean ± SD	0.1 ± 0.1	0.1 ± 0.1	0.1 ± 0.1	−0.05	**−0.10**	0.0	0.0
Electronic frailty index score, *n* (%)							
< 5%	359,778 (33.2)	808,898 (33.0)	268,084 (34.9)	0.01	−0.02	0.0	0.0
≥ 5% to < 20%	621,538 (57.4)	1,371,826 (56.0)	399,392 (52.0)	0.01	0.08	0.0	**0.1**
≥ 20%	102,150 (9.4)	267,679 (10.9)	100,249 (13.1)	−0.04	−0.09	0.0	0.0
Cardiovascular risk, *n* (%)							
Hospitalizations							
Myocardial infarction	4549 (0.4)	12,744 (0.5)	4726 (0.6)	−0.01	−0.03	0.0	0.0
Ischemic stroke	4694 (0.4)	12,644 (0.5)	5079 (0.7)	−0.01	−0.03	0.0	0.0
Heart failure	8303 (0.8)	24,277 (1.0)	9200 (1.2)	−0.03	−0.04	0.0	0.0
Transient ischemic attack	2013 (0.2)	4618 (0.2)	1808 (0.2)	0.00	−0.01	0.0	0.0
Hypercholesterolemia	161,022 (14.9)	378,064 (15.4)	123,956 (16.1)	0.00	0.00	0.0	0.0
Hypertension	660,031 (60.9)	1,570,173 (64.1)	526,956 (68.6)	−0.03	−0.10	0.0	0.0
Type 2 diabetes	285,306 (26.3)	699,691 (28.6)	257,683 (33.6)	−0.04	**−0.15**	0.0	0.0
Tobacco use, *n* (%)							
Current	13,203 (1.2)	36,928 (1.5)	11,785 (1.5)	−0.02	−0.02	0.0	0.0
Former	24,430 (2.3)	60,192 (2.5)	17,748 (2.3)	−0.01	0.01	0.0	0.0
Never	22,314 (2.1)	52,701 (2.2)	16,657 (2.2)	−0.01	0.00	0.0	0.0
Unknown/not reported	1,023,519 (94.5)	2,298,582 (93.9)	721,535 (94.0)	0.02	0.01	0.0	0.0
BMI, mean kg/m^2^ ± SD	28.7 ± 6.0	29.1 ± 6.1	29.1 ± 6.2				
BMI category, *n* (%)^b^							
< 18.5	5235 (0.5)	13,998 (0.6)	4746 (0.6)	−0.01	−0.01	0.0	0.0
18.5–24.9	82,417 (7.6)	216,119 (8.8)	67,073 (8.7)	−0.03	−0.02	**−0.1**	**−0.1**
25–29.9	124,043 (11.4)	330,565 (13.5)	102,924 (13.4)	−0.04	−0.02	**−0.1**	**−0.1**
≥ 30	124,576 (11.5)	363,632 (14.9)	115,802 (15.1)	−0.07	−0.06	0.0	0.0

Abbreviations: aIIV3, adjuvanted trivalent influenza vaccine; HD‐IIV3e, high‐dose, egg‐based trivalent influenza vaccine; IIV4e, egg‐based quadrivalent influenza vaccine; IPTW, inverse probability of treatment weighting; SD, standard difference; SMD, standard mean difference.

^a^ Boldface = statistically significant difference based on an absolute SMD value ≥ 0.1.

^b^ Percentages for BMI index do not sum to 100% because the data to calculate BMI were not available for all subjects.

### 3.2 Cardiorespiratory Hospitalization—Any Diagnosis Position

**FIGURE 1:**
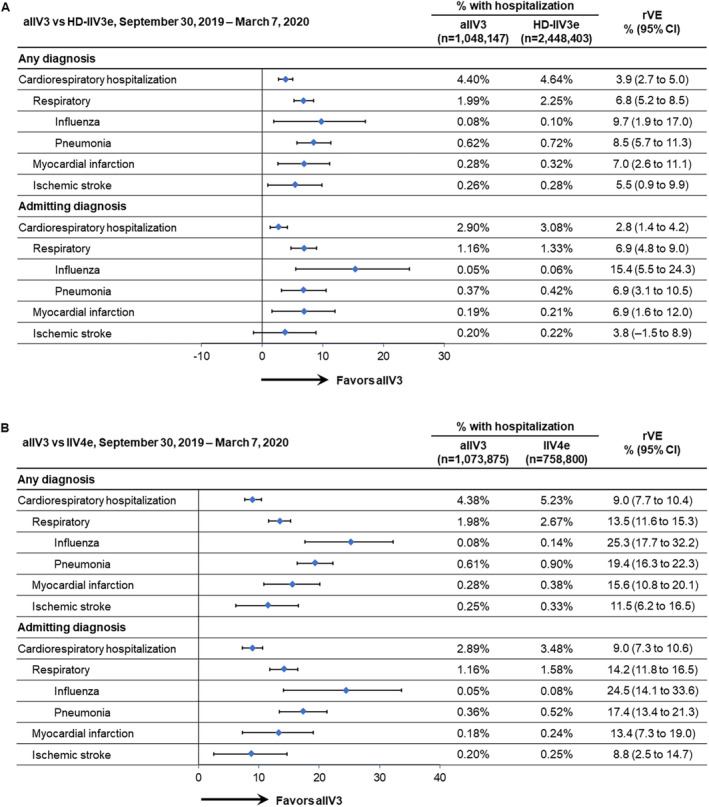
Adjusted relative vaccine effectiveness (rVE) between September 30, 2019, and March 7, 2020, of (A) adjuvanted trivalent influenza vaccine (aIIV3) versus high‐dose, egg‐based trivalent influenza vaccine (HD‐IIV3e) and (B) aIIV3 versus egg‐based quadrivalent influenza vaccine (IIV4e). CI, confidence interval.

After the IPTW‐weighted sample was truncated to remove extreme outlier subjects, the respective cohorts included 1,048,147 and 2,448,403 subjects for the comparison between aIIV3 and HD‐IIV3e and 1,073,875 and 758,800 subjects for the aIIV3 versus IIV4e comparison. Through March 7, 2020, 4.4%, 4.6%, and 5.2% of the aIIV3, HD‐IIV3e, and IIV4e cohorts were hospitalized for a cardiorespiratory diagnosis, of which approximately half were respiratory hospitalizations (Tables S5 and S6). Rates of hospitalizations for MI and stroke were similar between cohorts (Figure 1). Figure S1 shows the rVEs from the unadjusted analyses.

Overall, aIIV3 was associated with fewer hospitalizations linked to a cardiorespiratory diagnosis in any position. The rVE for aIIV3 versus HD‐IIV3 was 3.9% (95% CI, 2.7–5.0) and for aIIV3 versus IIV4e was 9.0% (95% CI, 7.7–10.4) for the overall cardiorespiratory endpoint. The point estimates were higher in the aIIV3 versus IIV4e than the aIIV3 versus HD‐IIV3e comparisons (Figure 1). In both comparisons, the highest point estimates were for influenza hospitalizations. The rVE of aIIV3 versus HD‐IIV3 against influenza hospitalizations was 9.7% (95% CI, 1.9–17.0) and for aIIV3 versus IIV4e was 25.3% (95% CI, 17.7–32.2). rVEs were statistically significant for all outcomes associated with a diagnosis in any position.

### 3.3 Cardiorespiratory Hospitalizations—Admitting Diagnostic Position

Relative to hospitalizations associated with the “any diagnosis” position, admitting diagnoses accounted for approximately two‐thirds fewer hospitalizations. The rVE patterns for the admitting diagnoses were similar to the patterns observed for outcomes associated with any diagnosis (Figure 1), with the highest point estimates for influenza hospitalizations in both the aIIV3 versus HD‐IIV3e and aIIV3 versus IIV4e comparisons. The rVE for aIIV3 versus IIV4e was higher in the admitting position although confidence intervals were wider. rVEs were statistically significant for all outcomes except for ischemic stroke in the aIIV3 versus HD‐IIV3e comparison, in which the 95% CI crossed the null (Figure 1).

### 3.4 Sensitivity Analyses

#### 3.4.1 Diagnoses in Any Position

**FIGURE 2:**
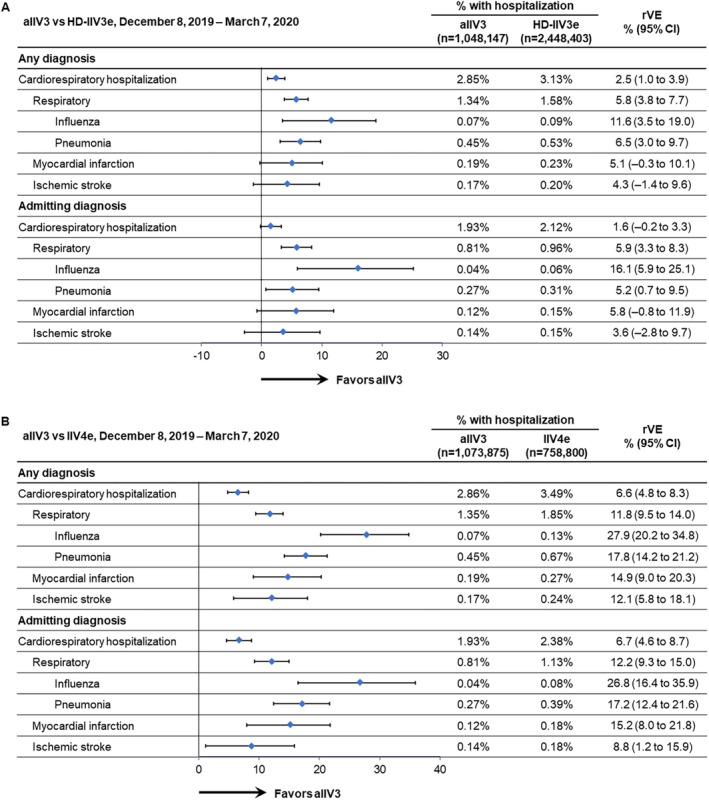
Adjusted relative vaccine effectiveness (rVE) between December 8, 2019, and March 10, 2020, of (A) adjuvanted trivalent influenza vaccine (aIIV3) versus high‐dose, egg‐based trivalent influenza vaccine (HD‐IIV3e) and (B) aIIV3 versus egg‐based quadrivalent influenza vaccine (IIV4e). CI, confidence interval.

During the 12‐week period of highest influenza activity (December 8, 2019, through March 7, 2020), the rVE of aIIV3 versus HD‐IIV3e for cardiorespiratory and respiratory hospitalizations, including those that were for influenza and pneumonia, was positive and statistically significant. The highest point estimates were for influenza hospitalizations. Point estimates for MI and stroke were also positive, but the 95% CIs crossed the null (Figure 2A). aIIV3 was more effective than IIV4e for prevention of all outcomes when the diagnosis was in any position (Figure 2B).

**FIGURE 3:**
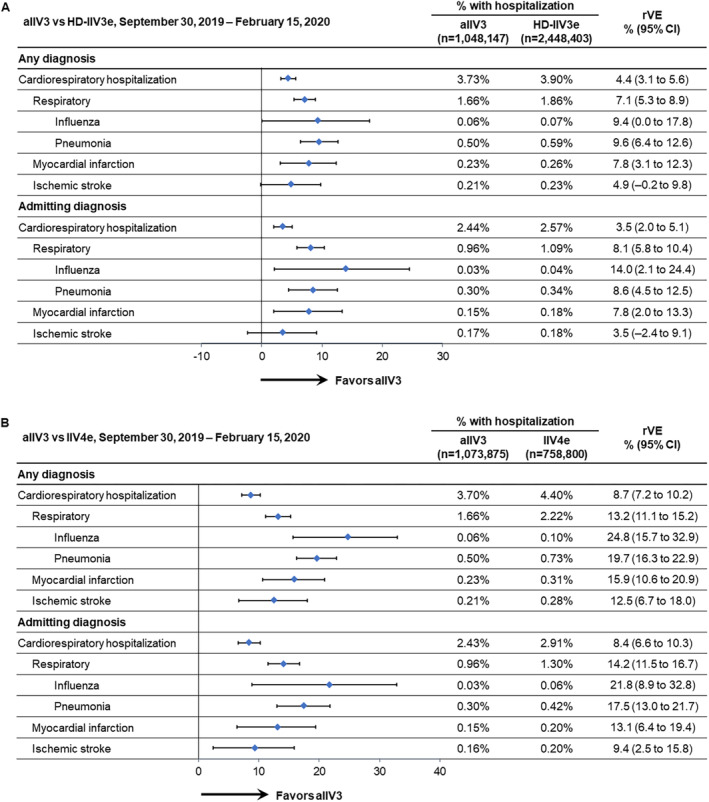
Adjusted relative vaccine effectiveness (rVE) between September 30, 2019, and February 15, 2020, of (A) adjuvanted trivalent influenza vaccine (aIIV3) versus high‐dose, egg‐based trivalent influenza vaccine (HD‐IIV3e) and (B) aIIV3 versus egg‐based quadrivalent influenza vaccine (IIV4e). CI, confidence interval.

Rates of hospitalizations and rVE patterns were similar to the main rVE analyses during the study period shortened to avoid confounding factors related to SARS‐CoV‐2 circulation (September 30, 2019, through February 15, 2020) (Figure 3). As in the main analysis, the stroke prevention rVE was not statistically significant in the aIIV3 versus HD‐IIV3e comparison, but rVEs were significant across the comparisons for all other outcomes, and the point estimates for prevention of influenza hospitalizations were the highest.

#### 3.4.2 Admitting Diagnoses

The point estimates for outcomes associated with an admitting diagnosis between December 8, 2019, and March 7, 2020 (peak influenza activity), were similar to those for any diagnosis in the comparisons between aIIV3 and HD‐IIV3e, and the rVE estimate for influenza hospitalizations was even higher than the rVE for any hospitalization with an influenza‐related diagnosis. However, only rVEs for respiratory hospitalizations (including influenza and pneumonia) were statistically significant (Figure 2A). In the comparisons between aIIV3 and IIV4e, the rVEs for all admitting diagnoses were positive and statistically significant (Figure 2B). Similar patterns were observed through February 15, 2020 (Figure 3).

### 3.5 Negative Control Outcomes

**FIGURE 4:**
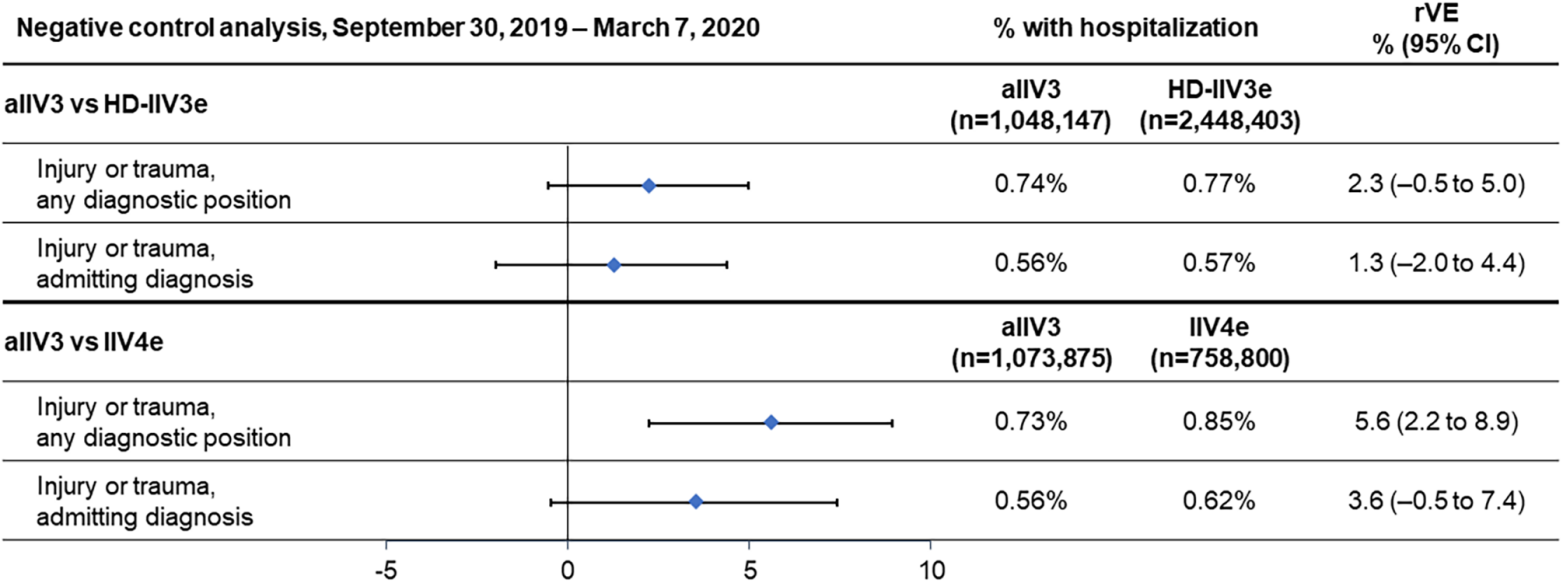
Negative control analysis comparing the incidence of hospitalizations for injury or trauma in each cohort the effect of vaccination with adjuvanted trivalent influenza vaccine (aIIV3), high‐dose, egg‐based trivalent influenza vaccine (HD‐IIV3e), and egg‐based quadrivalent influenza vaccine (IIV4e) between September 30, 2019, and March 7, 2020. CI, confidence interval; rVE, adjusted relative vaccine effectiveness.

Hospitalizations involving an injury or trauma in any diagnostic position were reported by 0.6% of each cohort and admissions for injury or trauma by 0.3% of each cohort. The negative control outcomes were similar between aIIV3 and HD‐IIV3e, but the point estimate and CI were above zero in the aIIV3 versus IIV4e comparison for injury or trauma in any diagnosis position (Figure 4).

In the post hoc analysis of additional negative control outcomes (appendicitis, cataracts, eyelid disorder, hemorrhoids, herpes zoster, lipoma, and nail disorders), hospitalization rates were less than 0.1% except for cataract‐related hospitalizations, which occurred at a rate close to 0.2%. Based on an analysis of adjusted rVEs, there were no detectable differences in the effectiveness of aIIV3 at preventing hospitalizations for the additional negative control outcomes compared to HD‐IIV3e or IIV4e, as most estimates were not significant, showing no clear trends favoring any of the evaluated vaccines (Table S7).

## 4 Discussion

In this study conducted during the 2019–2020 US influenza season, aIIV3 was associated with lower proportion of cardiorespiratory hospitalizations among adults ≥ 65 years of age compared with HD‐IIV3e and IIV4e. Subcategories of cardiorespiratory outcomes, including hospitalizations for respiratory virus infections overall, influenza, pneumonia, and MIs, were also reduced in both comparisons. aIIV3 was also associated with lower proportion of ischemic stroke hospitalizations when compared to IIV4e. Across nearly all comparisons and study periods, the rVE point estimates were highest for influenza hospitalizations, and protection against pneumonia or overall respiratory hospitalizations also favored aIIV3 versus the comparators. aIIV3 provided significant protection from hospitalizations for MI in any diagnosis position and as the admitting diagnosis relative to both comparators.

Although absolute differences in outcome proportions and rVEs in some comparisons may seem modest, the magnitude of burden averted becomes significant when considering the vast number of individuals vaccinated and the higher burden of noninfluenza‐specific events such as MI and stroke‐related hospitalizations [35, 36]. Assuming absolute vaccine effectiveness of 39% reported by the CDC is reflective of HD‐IIV3e, given the majority of influenza coverage involves HD‐IIV3e, we can calculate the absolute vaccine effectiveness of aIIV3 against influenza‐related hospitalizations as rVE × (1 − VE (HD‐IIV3e)) + aVE (HD‐IIV3e), which yields 44.9%. Assuming an aVE of 5% for HD‐IIV3e against cardiovascular hospitalizations, the aVE of aIIV3 would be 5.2%. In addition to rVE, estimating absolute vaccine effectiveness (aVE) further contributes to the understanding of the overall impact of vaccination with aIIV3.

The 2019–2020 influenza season consisted of two waves, first of influenza B, followed by A(H1N1). Influenza A(H1N1) was the predominant strain in the older adult population and accounted for 73% of all circulating viruses. Absolute vaccine effectiveness was estimated to be 39% (95% CI, 32–44) overall and 39% (95% CI, 9–59) against medically attended outpatient influenza in adults age ≥ 65 years by the CDC [37].

A recent meta‐analysis of 16 studies assessing the effect of influenza vaccination on cardiovascular outcomes among mostly older adult populations with high cardiovascular risk factors demonstrated that vaccination against influenza reduced the relative risk of all‐cause mortality by 25% (risk ratio [RR], 0.75 [95% CI, 0.60–0.93; *p* = 0.01]), cardiovascular mortality by 18% (RR, 0.82 [95% CI, 0.80–0.84; *p* < 0.001]), and major adverse cardiovascular events by 13% (RR, 0.87 [95% CI, 0.80–0.94; *p* < 0.001]) [38]. The magnitude and breadth of the immune response is increased by MF59 compared with traditional inactivated influenza vaccines, which may explain the greater effectiveness of aIIV3 over IIV4e observed in this and other studies [16, 17, 18, 19, 20, 39].

Viewed in light of the current evidence, most prior studies have shown comparability in the effectiveness of aIIV3 versus HD‐IIV3 in preventing influenza‐related medical encounters in adults aged ≥ 65 years [40]. As HD‐IIV3e also increases the magnitude of the immune response, the relative benefits of aIIV3 versus HD‐IIV3 observed in this study may be due to the breadth of the immune response offered by aIIV3 [41]. This difference between aIIV3 and HD‐IIV3 may explain the relative benefits of aIIV3 versus HD‐IIV3 observed in this study.

Other studies specifically examining the effect of aIIV3 on hospitalizations for influenza, pneumonia, and cardiovascular outcomes have shown that the addition of an adjuvant, and the corresponding boost in the immune response, confers a protective effect on older adults [16, 17, 18, 20]. Our findings are consistent with these results.

This study has several limitations. First, variability in how individuals utilize US healthcare resources and inherent heterogeneity in the healthcare system is a potential source of bias. Subject utilization of healthcare resources may be intermittent or opportunistic, and the amount and quality of data available on individuals may vary. This is unlikely to affect hospitalization outcomes, but variations in the amount and quality of available data may affect the balancing of subjects by baseline characteristics. Second, we could not confirm whether influenza‐related events were associated laboratory‐confirmed influenza cases, which might lead to outcome misclassification. However, the peak influenza season, or MEM analysis, aimed to improve outcome specificity in the absence of laboratory confirmation of influenza. Findings were very similar in terms of both magnitude and direction between the full outcome assessment period (September 30, 2019, to March 7, 2020) and the peak influenza season (December 8, 2019, to March 7, 2020). Patterns were also similar when the study period was shortened further to February 15, 2020, to avoid outcome misclassification due to SARS‐CoV‐2 co‐circulation. Furthermore, cardiorespiratory hospitalizations may occur as a result of downstream complications of influenza virus infection, even after a subject has recovered and may no longer test positive for influenza, making laboratory confirmation for these outcomes irrelevant. Third, vaccination was not randomly assigned, and unmeasured confounding might bias estimates. Residual confounding is a potential source of bias in all observational research; however, it is particularly prominent in studies using routinely collected data as these data are not specifically collected for research purposes.

Nevertheless, the generalizability of this research is supported by the very large sample size including only subjects active within the healthcare system, which mitigated against bias due to small sample size and provided ample statistical power, minimizing bias in rVE point estimates and CIs. The broad geographical extent of the dataset further minimized regional differences in the sample, and the subject demographics are similar to the overall US population. The integrated dataset combines the clinical details of EMR data with the comprehensive care details of claims data. Exposure, outcome, and covariate information were ascertained similarly across all exposure cohorts, limiting the possibility of differential misclassification. Finally, the IPTW approach mitigated demographic and clinical heterogeneity between vaccination groups. The adjusted cohorts were balanced across demographic confounders to weight out differences across all subjects and vaccine types; this approach blunted any critical differences in demographic biases across vaccine types.

In conclusion, adults aged ≥ 65 years vaccinated with aIIV3 during the 2019–2020 influenza season experienced fewer cardiorespiratory hospitalizations—including those for respiratory virus infections overall, influenza, pneumonia, and MIs—than older adults who were vaccinated with HD‐IIV3e or IIV4e. In addition, fewer hospitalizations for ischemic stroke were reported among older adults who received aIIV3 than in those receiving IIV4e.

## Author Contributions

**Mahrukh Imran:** Conceptualization (lead); Investigation (lead); Methodology; Project administration; Resources (lead); Supervision (lead); Writing – original draft (lead); Writing – review and editing. **Joan Puig‐Barbera:** Methodology; Writing – review and editing. **Justin R. Ortiz:** Investigation; Writing – review and editing. **Lorena Lopez‐Gonzalez:** Data curation; Formal analysis; Project administration; Writing – review and editing. **Alex Dean:** Data curation. **Machaon Bonafede:** Formal analysis; Methodology; Supervision; Writing – review and editing. **Mendel Haag:** Conceptualization; Funding acquisition; Methodology; Resources; Software; Supervision; Writing – review and editing.

## Conflicts of Interest

M.I. and M.H. are employees of CSL Seqirus. J.P. has received honoraria as adviser, research activities, or conferences from Hipra, Novavax, Sanofi, and Seqirus. J.R.O. reports that his institution has received research grants from National Institutes of Health for influenza vaccine research. He has received honoraria from GSK, Moderna, Pfizer, and Seqirus to serve on scientific advisory boards. L.L.G. was an employee of Veradigm during the analysis execution and is currently an employee of BioCryst Pharmaceuticals, Inc. A.D. and M.B. are employees of Veradigm.

## Supporting information


**Table S1.** Influenza vaccine codes.
**Table S2.** ICD‐10‐CM diagnostic codes used to identify hospitalizations related to cardiorespiratory diseases.
**Table S3.** Week of vaccination.^a^
**Table S4.** Subjects with individual comorbidities included in the Charlson Comorbidity Index at baseline.^1,2^
**Table S5.** Unweighted and unadjusted rVEs for cardiorespiratory hospitalizations and for the negative control outcome of injury/trauma during the 2019–2020 influenza season (September 30, 2019–March 7, 2020) among older adults aged ≥ 65 years.
**Table S6.** Weighted and adjusted rVEs for cardiorespiratory hospitalizations and for the negative control outcome of injury/trauma during the 2019–2020 influenza season (September 30, 2019–March 7, 2020) among older adults aged ≥ 65 years.
**Figure S1.** Unadjusted relative vaccine effectiveness (rVE) of (A) adjuvanted trivalent influenza vaccine (aIIV3) versus high‐dose, egg‐based trivalent influenza vaccine (HD‐IIV3e) and (B) aIIV3 versus egg‐based quadrivalent influenza vaccine (IIV4e) between September 30, 2019, and March 7, 2020. CI, confidence interval.
**Table S7.** Analyses of additional negative control outcomes between September 30, 2019, and March 7, 2020.

## Data Availability

The datasets used in this study are privately licensed and are not available to be shared publicly.
