# Supplementary material for: Relative Effectiveness of the MF59®‐Adjuvanted Influenza Vaccine Versus High‐Dose and Non‐Adjuvanted Influenza Vaccines in Preventing Cardiorespiratory Hospitalizations During the 2019–2020 US Influenza Season
Source: Influenza Other Respir Viruses. 2024 Apr 21;18(4):e13288. doi: 10.1111/irv.13288 (PMC11033326; doi:10.1111/irv.13288)
Supplement: Supplementary file 1 — Table S1. Influenza vaccine codes. Table S2. ICD‐10‐CM diagnostic codes used to identify hospitalizations related to cardiorespiratory diseases. Table S3. Week of vaccination.a Table S4. Subjects with individual comorbidities included in the Charlson Comorbidity Index at baseline.1,2 Table S5. Unweighted and unadjusted rVEs for cardiorespiratory hospitalizations and for the negative control outcome of injury/trauma during the 2019–2020 influenza season (September 30, 2019–March 7, 2020) among older adults aged ≥ 65 years. Table S6. Weighted and adjusted rVEs for cardiorespiratory hospitalizations and for the negative control outcome of injury/trauma during the 2019–2020 influenza season (September 30, 2019–March 7, 2020) among older adults aged ≥ 65 years. Figure S1. Unadjusted relative vaccine effectiveness (rVE) of (A) adjuvanted trivalent influenza vaccine (aIIV3) versus high‐dose, egg‐based trivalent influenza vaccine (HD‐IIV3e) and (B) aIIV3 versus egg‐based quadrivalent influenza vaccine (IIV4e) between September 30, 2019, and March 7, 2020. CI, confidence interval. Table S7. Analyses of additional negative control outcomes between September 30, 2019, and March 7, 2020. [file IRV-18-e13288-s001.docx]

Supplement to:

Relative effectiveness of the MF-59^®^-adjuvanted influenza vaccine vs. high-dose and standard influenza vaccine in preventing cardiorespiratory hospitalizations during the 2019–2020 US influenza season

Mahrukh Imran, Juan Puig-Barbera, Justin R. Ortiz, Lorena Lopez-Gonzalez, Alex Dean, Machaon Bonafede^4^, Mendel Haag^5^

Table S1. Influenza vaccine codes.

| **Vaccine type** | **Code type** | **Code** | **Description** | **Manufacturer** | **Year** |
| --- | --- | --- | --- | --- | --- |
| Adjuvanted | NDC | 70461001904 | FLUAD | Seqirus | 2019 |
| Adjuvanted | NDC | 70461001903 | FLUAD | Seqirus | 2019 |
| Adjuvanted | CPT | 90653 | Vaccine for influenza for injection into muscle, inactivated, subunit, adjuvanted | n/a | n/a |
| Adjuvanted | CPT | 90689 | IIV4, inactivated, adjuvanted, preservative free, 0.25mL dosage, for intramuscular use |  | n/a |
| Adjuvanted | CVX | 168 | Influenza, trivalent, adjuvanted | n/a | n/a |
| High Dose | NDC | 49281040565 | FLUZONE High-Dose (quadrivalent) | Sanofi | 2019 |
| High Dose | CVX | 135 | Influenza, high dose seasonal, preservative-free |  | n/a |
| High Dose | CPT | 90662 | IIV4, split virus, preservative free, enhanced immunogenicity via increased antigen content, for intramuscular use |  | n/a |
| High Dose | NDC | 49281040588 | FLUZONE High-Dose (quadrivalent) | Sanofi | 2019 |
| High Dose | CVX | 197 | Influenza, high-dose, quadrivalent |  | n/a |
| IIV4e | CVX | 161 | Influenza, injectable, quadrivalent, preservative free, pediatric | n/a | n/a |
| IIV4e | CPT | 90685 | IIV4, split virus, preservative free, 0.25 mL dosage, for intramuscular use | n/a | n/a |
| IIV4e | NDC | 19515089701 | Flulaval Quadrivalent 2019–2020 | GSK | 2019 |
| IIV4e | NDC | 49281041950 | Fluzone Quadrivalent 2019–2020 | Sanofi | 2019 |
| IIV4e | NDC | 33332041911 | Afluria Quadrivalent 2019–2020 | Seqirus | 2019 |
| IIV4e | NDC | 58160089652 | Fluarix Quadrivalent 2019–2020 | GSK | 2019 |
| IIV4e | CPT | 90688 | IIV4, split virus, 0.5 mL dosage, for intramuscular use | n/a | n/a |
| IIV4e | NDC | 33332021921 | Afluria Quadrivalent 2019–2020 | Seqirus | 2019 |
| IIV4e | NDC | 49281041910 | Fluzone Quadrivalent 2019–2020 | Sanofi | 2019 |
| IIV4e | CVX | 166 | Influenza, intradermal, quadrivalent, preservative free | n/a | n/a |
| IIV4e | CPT | 90686 | IIV4, split virus, preservative free, 0.5 mL dosage, for intramuscular use | n/a | n/a |
| IIV4e | NDC | 49281051900 | Fluzone Quadrivalent 2019–2020 | Sanofi | 2019 |
| IIV4e | NDC | 49281063115 | Fluzone Quadrivalent 2019–2020 | Sanofi | 2019 |
| IIV4e | NDC | 19515090641 | Flulaval Quadrivalent 2019–2020 | GSK | 2019 |
| IIV4e | NDC | 33332031902 | Afluria Quadrivalent 2019–2020 | Seqirus | 2019 |
| IIV4e | NDC | 33332031901 | Afluria Quadrivalent 2019–2020 | Seqirus | 2019 |
| IIV4e | NDC | 33332021920 | Afluria Quadrivalent 2019–2020 | Seqirus | 2019 |
| IIV4e | CVX | 158 | Influenza, injectable, quadrivalent, contains preservative | n/a | n/a |
| IIV4e | CVX | 150 | Influenza, injectable, quadrivalent, preservative free | n/a | n/a |
| IIV4e | NDC | 49281041988 | Fluzone Quadrivalent 2019–2020 | Sanofi | 2019 |
| IIV4e | NDC | 33332041910 | Afluria Quadrivalent 2019–2020 | Seqirus | 2019 |
| IIV4e | NDC | 49281041958 | Fluzone Quadrivalent 2019–2020 | Sanofi | 2019 |
| IIV4e | CPT | 90630 | IIV4, split virus, preservative free, for intradermal use | n/a | n/a |
| IIV4e | NDC | 49281051925 | Fluzone Quadrivalent 2019–2020 | Sanofi | 2019 |
| IIV4e | NDC | 19515090652 | Flulaval Quadrivalent 2019–2020 | GSK | 2019 |
| IIV4e | CPT | 90687 | IIV4, split virus, 0.25 mL dosage, for intramuscular use | n/a | n/a |
| IIV4e | NDC | 19515089711 | Flulaval Quadrivalent 2019–2020 | GSK | 2019 |
| IIV4e | NDC | 58160089641 | Fluarix Quadrivalent 2019–2020 | GSK | 2019 |
| IIV4e | NDC | 49281063178 | Fluzone Quadrivalent 2019–2020 | Sanofi | 2019 |

CVX, codes for vaccines administered; CPT, Current Procedural Terminology; HA, hemagglutinin; IIV4, quadrivalent influenza virus vaccine; NDC, national drug code

Table S2. ICD-10-CM diagnostic codes used to identify hospitalizations related to cardiorespiratory diseases.

| **Study Outcome** | **ICD-10-CM** |
| --- | --- |
| Cardiorespiratory hospitalization | Ixx – Jxx |
| Respiratory hospitalization | Jxx |
| Pneumonia hospitalization | J12x – J18x |
| Influenza hospitalization | J09x – J11x |
| Hospitalization for myocardial infarction | I21x – I23x |
| Hospitalization for ischemic stroke | I63x |
| **Negative control outcome** |  |
| Injury/trauma hospitalization | S00x – T32x |

Table S3. Week of vaccination.^a^

|  | | **EHR and full claims** | | | | | |
| --- | --- | --- | --- | --- | --- | --- | --- |
|  |  | **aIIV3**  **(n=1,083,466)** | | **HD-IIV3e**  **(n=2,448,403)** | | **IIV4e**  **(n=767,725)** | |
| **Week starting** | **Week ending** | **n** | **%** | **n** | **%** | **n** | **%** |
| 7/28/2019 | 8/3/2019 | 364 | 0.0% | 508 | 0.0% | 332 | 0.0% |
| 8/4/2019 | 8/10/2019 | 6,850 | 0.6% | 311 | 0.0% | 1,139 | 0.2% |
| 8/11/2019 | 8/17/2019 | 14,689 | 1.4% | 616 | 0.0% | 2,580 | 0.3% |
| 8/18/2019 | 8/24/2019 | 22,827 | 2.1% | 4,204 | 0.2% | 4,663 | 0.6% |
| 8/25/2019 | 8/31/2019 | 26,740 | 2.5% | 21,579 | 0.9% | 8,124 | 1.1% |
| 9/1/2019 | 9/7/2019 | 35,180 | 3.3% | 57,363 | 2.3% | 17,090 | 2.2% |
| 9/8/2019 | 9/14/2019 | 64,279 | 5.9% | 101,279 | 4.1% | 33,626 | 4.4% |
| 9/15/2019 | 9/21/2019 | 80,355 | 7.4% | 172,667 | 7.1% | 49,048 | 6.4% |
| 9/22/2019 | 9/28/2019 | 96,708 | 8.9% | 234,361 | 9.6% | 64,257 | 8.4% |
| 9/29/2019 | 10/5/2019 | 118,560 | 10.9% | 303,020 | 12.4% | 88,838 | 11.6% |
| 10/6/2019 | 10/12/2019 | 138,929 | 12.8% | 272,497 | 11.1% | 98,443 | 12.8% |
| 10/13/2019 | 10/19/2019 | 110,098 | 10.2% | 232,925 | 9.5% | 93,486 | 12.2% |
| 10/20/2019 | 10/26/2019 | 90,240 | 8.3% | 240,579 | 9.8% | 73,244 | 9.5% |
| 10/27/2019 | 11/2/2019 | 75,164 | 6.9% | 175,478 | 7.2% | 52,295 | 6.8% |
| 11/3/2019 | 11/9/2019 | 53,898 | 5.0% | 145,709 | 6.0% | 39,193 | 5.1% |
| 11/10/2019 | 11/16/2019 | 37,871 | 3.5% | 111,517 | 4.6% | 29,235 | 3.8% |
| 11/17/2019 | 11/23/2019 | 30,842 | 2.9% | 93,265 | 3.8% | 24,716 | 3.2% |
| 11/24/2019 | 11/30/2019 | 14,182 | 1.3% | 42,626 | 1.7% | 11,389 | 1.5% |
| 12/1/2019 | 12/7/2019 | 16,868 | 1.6% | 56,745 | 2.3% | 15,212 | 2.0% |
| 12/8/2019 | 12/14/2019 | 13,524 | 1.3% | 48,801 | 2.0% | 13,553 | 1.8% |
| 12/15/2019 | 12/21/2019 | 10,160 | 0.9% | 36,056 | 1.5% | 10,428 | 1.4% |
| 12/22/2019 | 12/28/2019 | 4,312 | 0.4% | 13,720 | 0.6% | 3,845 | 0.5% |
| 12/29/2019 | 1/4/2020 | 4,446 | 0.4% | 16,236 | 0.7% | 5,396 | 0.7% |
| 1/5/2020 | 1/11/2020 | 5,778 | 0.5% | 23,139 | 1.0% | 9,162 | 1.2% |
| 1/12/2020 | 1/18/2020 | 4,598 | 0.4% | 17,918 | 0.7% | 7,559 | 1.0% |
| 1/19/2020 | 1/25/2020 | 3,244 | 0.3% | 13,363 | 0.6% | 5,794 | 0.8% |
| 1/26/2020 | 2/1/2020 | 2,760 | 0.3% | 11,921 | 0.5% | 5,078 | 0.7% |

^a^The vaccination identification period was defined as August 1, 2019, through January 31, 2020.

aIIV3, adjuvanted trivalent influenza vaccine; EHR, electronic health record; HD-IIV3e, high-dose, egg-based trivalent influenza vaccine; IIV4e, egg-based quadrivalent influenza vaccine.

Table S4. Subjects with individual comorbidities included in the Charlson Comorbidity Index at baseline.^1,2^

| Number (%) | aIIV3 (n=1,083,466) | HD-IIV3e (n=2,448,403) | IIV4e (n=767,725) | IPTW SMD | |
| --- | --- | --- | --- | --- | --- |
|  |  |  |  | aIIV3 vs HD-IIV3e | aIIV3 vs IIV4e |
| Myocardial infarction | 38,316 (3.5) | 99,600 (4.1) | 34,350 (4.5) | 0.0 | 0.0 |
| Congestive heart failure | 100,873 (9.3) | 263,918 (10.8) | 95,633 (12.5) | 0.0 | 0.0 |
| Peripheral vascular disease | 150,032 (13.8) | 359,279 (14.7) | 139,864 (18.2) | 0.0 | 0.0 |
| Cerebrovascular disease | 104,173 (9.6) | 244,194 (10.0) | 83,993 (10.9) | 0.0 | 0.0 |
| Dementia | 44,590 (4.1) | 119,298 (4.9) | 52,127 (6.8) | 0.0 | 0.0 |
| Chronic pulmonary disease | 194,592 (18.0) | 472,636 (19.3) | 168,591 (22.0) | 0.0 | 0.0 |
| Rheumatic disease | 42,417 (3.9) | 98,594 (4.0) | 33,317 (4.3) | 0.0 | 0.0 |
| Peptic ulcer disease | 12,161 (1.1) | 28,684 (1.2) | 10,804 (1.4) | 0.0 | 0.0 |
| Liver disease, mild | 44,270 (4.1) | 105,638 (4.3) | 42,290 (5.5) | 0.0 | 0.0 |
| Diabetes without chronic complications | 268,549 (24.8) | 659,579 (26.9) | 244,390 (31.8) | 0.0 | 0.0 |
| Renal disease, mild to moderate | 113,673 (10.5) | 286,374 (11.7) | 101,784 (13.3) | 0.0 | 0.0 |
| Diabetes with chronic complications | 136,092 (12.6) | 348,404 (14.2) | 130,692 (17.0) | 0.0 | 0.0 |
| Hemiplegia or paraplegia | 6,451 (0.6) | 18,721 (0.8) | 8,496 (1.1) | 0.1 | 0.0 |
| Any malignancy | 110,930 (10.2) | 255,239 (10.4) | 76,297 (9.9) | 0.0 | 0.0 |
| Liver disease, moderate to severe | 3,218 (0.3) | 8,379 (0.3) | 3,467 (0.5) | 0.0 | 0.0 |
| Renal disease, severe | 10,528 (1.0) | 44,970 (1.8) | 13,824 (1.8) | -0.1 | 0.0 |
| HIV infection, no AIDS | 1,235 (0.1) | 2,878 (0.1) | 2,034 (0.3) | 0.0 | 0.0 |
| Metastatic solid tumor | 10,621 (1.0) | 26,206 (1.1) | 8,337 (1.1) | 0.0 | 0.0 |
| AIDS | 159 (0.0) | 428 (0.0) | 261 (0.0) | 0.0 | 0.0 |

Boldface = statistically significant difference based on an absolute SMD value ≥0.1.

aIIV3, adjuvanted trivalent influenza vaccine; HD-IIV3e, high-dose, egg-based trivalent influenza vaccine; IIV4e, egg-based quadrivalent influenza vaccine; IPTW, inverse probability of treatment weighting; SMD, standard mean difference.

1. Quan H, Sundararajan V, Halfon P, Fong A, Burnand B, Luthi JC, Saunders LD, Beck CA, Feasby TE, Ghali WA. Coding algorithms for defining comorbidities in ICD-9-CM and ICD-10 administrative data. Med Care 2005;43:1130-1139. doi: 10.1097/01.mlr.0000182534.19832.83. PMID: 16224307.

2. Sundararajan V, Henderson T, Perry C, Muggivan A, Quan H, Ghali WA. New ICD-10 version of the Charlson comorbidity index predicted in-hospital mortality. J Clin Epidemiol 2004;57:1288-1294. doi: 10.1016/j.jclinepi.2004.03.012. PMID: 15617955

Table S5. Unweighted and unadjusted rVEs for cardiorespiratory hospitalizations and for the negative control outcome of injury/trauma during the 2019–2020 influenza season (September 30, 2019 – March 7, 2020) among older adults aged ≥65 years

|  | **aIIV3 vs. HD-IIV3e** | | | | | | | **aIIV3 vs. IIV4e** | | | | | | |
| --- | --- | --- | --- | --- | --- | --- | --- | --- | --- | --- | --- | --- | --- | --- |
|  | **aIIV3**  **(n=1,083,466)** | | **HD-IIV3e**  **(n=2,448,403)** | | **rVE (%)** | **95% CI** | | **aIIV3**  **(n=1,083,466)** | | **IIV4e**  **(n=767,725)** | | **rVE (%)** | **95% CI** | |
|  | **n** | **%** | **n** | **%** |  | **Lower** | **Upper** | **n** | **%** | **n** | **%** |  | **Lower** | **Upper** |
| **Hospitalization outcomes, diagnosis in any position** | | | | | | | | | | | | | | |
| Cardiorespiratory hospitalization | 47,683 | 4.4% | 113,669 | 4.6% | 5.7 | 4.7 | 6.7 | 47,683 | 4.4% | 40,021 | 5.2% | 17.0 | 15.8 | 18.1 |
| Respiratory-related hospitalization | 21,667 | 2.0% | 55,018 | 2.2% | 11.3 | 9.9 | 12.7 | 21,667 | 2.0% | 20,386 | 2.7% | 25.4 | 24.0 | 26.9 |
| Influenza-related hospitalization | 895 | 0.1% | 2,406 | 0.1% | 16.0 | 9.2 | 22.2 | 895 | 0.1% | 1,054 | 0.1% | 39.9 | 34.3 | 45.0 |
| Pneumonia-related hospitalization | 6,716 | 0.6% | 17,697 | 0.7% | 14.4 | 11.9 | 16.7 | 6,716 | 0.6% | 6,900 | 0.9% | 31.3 | 28.9 | 33.6 |
| Hospitalization for myocardial infarction | 3,011 | 0.3% | 7,823 | 0.3% | 13.1 | 9.3 | 16.7 | 3,011 | 0.3% | 2,923 | 0.4% | 27.1 | 23.3 | 30.7 |
| Hospitalization for ischemic stroke | 2,782 | 0.3% | 6,920 | 0.3% | 9.2 | 5.1 | 13.1 | 2,782 | 0.3% | 2,560 | 0.3% | 23.1 | 18.9 | 27.1 |
|  |  |  |  |  |  |  |  |  |  |  |  |  |  |  |
| Injury/trauma-related hospitalization | 7,949 | 0.7% | 18,783 | 0.8% | 4.4 | 1.9 | 6.9 | 7,949 | 0.7% | 6,566 | 0.9% | 14.4 | 11.6 | 17.2 |
| **Hospitalization outcomes, primary/admitting diagnosis** | | | | | | | | | | | | | | |
| Cardiorespiratory hospitalization | 31,487 | 2.9% | 75,324 | 3.1% | 6.0 | 4.7 | 7.2 | 31,487 | 2.9% | 26,628 | 3.5% | 17.4 | 16.0 | 18.7 |
| Respiratory-related hospitalization | 12,686 | 1.2% | 32,541 | 1.3% | 12.1 | 10.3 | 13.9 | 12,686 | 1.2% | 12,088 | 1.6% | 26.2 | 24.3 | 28.0 |
| Influenza-related hospitalization | 501 | 0.0% | 1,447 | 0.1% | 21.8 | 13.4 | 29.3 | 501 | 0.0% | 606 | 0.1% | 41.4 | 34.1 | 48.0 |
| Pneumonia-related hospitalization | 3,982 | 0.4% | 10,308 | 0.4% | 12.8 | 9.5 | 15.9 | 3,982 | 0.4% | 3,973 | 0.5% | 29.2 | 26.0 | 32.2 |
| Hospitalization for myocardial infarction | 2,010 | 0.2% | 5,156 | 0.2% | 11.9 | 7.3 | 16.4 | 2,010 | 0.2% | 1,875 | 0.2% | 24.1 | 19.2 | 28.8 |
| Hospitalization for ischemic stroke | 2,173 | 0.2% | 5,277 | 0.2% | 7.0 | 2.2 | 11.5 | 2,173 | 0.2% | 1,901 | 0.2% | 19.1 | 13.9 | 23.9 |
|  |  |  |  |  |  |  |  |  |  |  |  |  |  |  |
| Injury/trauma-related hospitalization | 6,067 | 0.6% | 13,987 | 0.6% | 2.0 | -1.0 | 5.0 | 6,067 | 0.6% | 4,792 | 0.6% | 10.5 | 7.0 | 13.8 |

aIIV3, adjuvanted trivalent influenza vaccine; CI, confidence interval; HD-IIV3e, high-dose, egg-based trivalent influenza vaccine; IIV4e, egg-based quadrivalent influenza vaccine; rVE, relative vaccine effectiveness.

Table S6. Weighted and adjusted rVEs for cardiorespiratory hospitalizations and for the negative control outcome of injury/trauma during the 2019–2020 influenza season (September 30, 2019 – March 7, 2020) among older adults aged ≥65 years

|  | **aIIV3 vs. HD-IIV3e** | | | | | | | **aIIV3 vs. IIV4e** | | | | | | |
| --- | --- | --- | --- | --- | --- | --- | --- | --- | --- | --- | --- | --- | --- | --- |
|  | **aIIV3**  **(n=1,048,147)** | | **HD-IIV3e**  **(n=2,448,403)** | | **rVE (%)** | **95% CI** | | **aIIV3**  **(n=1,073,875)** | | **IIV4e**  **(n=758,800)** | | **rVE (%)** | **95% CI** | |
|  | **n** | **%** | **n** | **%** |  | **Lower** | **Upper** | **n** | **%** | **n** | **%** |  | **Lower** | **Upper** |
| **Hospitalization outcomes, diagnosis in any position** | | | | | | | | | | | | | | |
| Cardiorespiratory hospitalization | 46,148 | 4.4% | 113,669 | 4.6% | 3.9 | 2.7 | 5.0 | 47,043 | 4.4% | 39,674 | 5.2% | 9.0 | 7.7 | 10.4 |
| Respiratory-related hospitalization | 20,837 | 2.0% | 55,018 | 2.2% | 6.8 | 5.2 | 8.5 | 21,308 | 2.0% | 20,234 | 2.7% | 13.5 | 11.6 | 15.3 |
| Influenza-related hospitalization | 848 | 0.1% | 2,406 | 0.1% | 9.7 | 1.9 | 17.0 | 869 | 0.1% | 1,051 | 0.1% | 25.3 | 17.7 | 32.2 |
| Pneumonia-related hospitalization | 6,457 | 0.6% | 17,697 | 0.7% | 8.5 | 5.7 | 11.3 | 6,591 | 0.6% | 6,843 | 0.9% | 19.4 | 16.3 | 22.3 |
| Hospitalization for myocardial infarction | 2,908 | 0.3% | 7,823 | 0.3% | 7.0 | 2.6 | 11.1 | 2,964 | 0.3% | 2,899 | 0.4% | 15.6 | 10.8 | 20.1 |
| Hospitalization for ischemic stroke | 2,692 | 0.3% | 6,920 | 0.3% | 5.5 | 0.9 | 9.9 | 2,726 | 0.3% | 2,530 | 0.3% | 11.5 | 6.2 | 16.5 |
|  |  |  |  |  |  |  |  |  |  |  |  |  |  |  |
| Injury/trauma-related hospitalization | 7,710 | 0.7% | 18,783 | 0.8% | 2.3 | -0.5 | 5.0 | 7,857 | 0.7% | 6,468 | 0.9% | 5.6 | 2.2 | 8.9 |
| **Hospitalization outcomes, primary/admitting diagnosis** | | | | | | | | | | | | | | |
| Cardiorespiratory hospitalization | 30,436 | 2.9% | 75,324 | 3.1% | 2.8 | 1.4 | 4.2 | 31,047 | 2.9% | 26,413 | 3.5% | 9.0 | 7.3 | 10.6 |
| Respiratory-related hospitalization | 12,202 | 1.2% | 32,541 | 1.3% | 6.9 | 4.8 | 9.0 | 12,468 | 1.2% | 12,000 | 1.6% | 14.2 | 11.8 | 16.5 |
| Influenza-related hospitalization | 475 | 0.0% | 1,447 | 0.1% | 15.4 | 5.5 | 24.3 | 488 | 0.0% | 606 | 0.1% | 24.5 | 14.1 | 33.6 |
| Pneumonia-related hospitalization | 3,831 | 0.4% | 10,308 | 0.4% | 6.9 | 3.1 | 10.5 | 3,908 | 0.4% | 3,942 | 0.5% | 17.4 | 13.4 | 21.3 |
| Hospitalization for myocardial infarction | 1,949 | 0.2% | 5,156 | 0.2% | 6.9 | 1.6 | 12.0 | 1,984 | 0.2% | 1,859 | 0.2% | 13.4 | 7.3 | 19.0 |
| Hospitalization for ischemic stroke | 2,108 | 0.2% | 5,277 | 0.2% | 3.8 | -1.5 | 8.9 | 2,132 | 0.2% | 1,875 | 0.2% | 8.8 | 2.5 | 14.7 |
|  |  |  |  |  |  |  |  |  |  |  |  |  |  |  |
| Injury/trauma-related hospitalization | 5,887 | 0.6% | 13,987 | 0.6% | 1.3 | -2.0 | 4.4 | 6,000 | 0.6% | 4,714 | 0.6% | 3.6 | -0.5 | 7.4 |

aIIV3, adjuvanted trivalent influenza vaccine; CI, confidence interval; HD-IIV3e, high-dose, egg-based trivalent influenza vaccine; IIV4e, egg-based quadrivalent influenza vaccine; rVE, relative vaccine effectiveness.

Figure S1. Unadjusted relative vaccine effectiveness (rVE) of (*A*) adjuvanted trivalent influenza vaccine (aIIV3) vs high-dose, egg-based trivalent influenza vaccine (HD-IIV3e) and (*B*) aIIV3 vs egg-based quadrivalent influenza vaccine (IIV4e) between September 30, 2019, and March 7, 2020. CI, confidence interval.


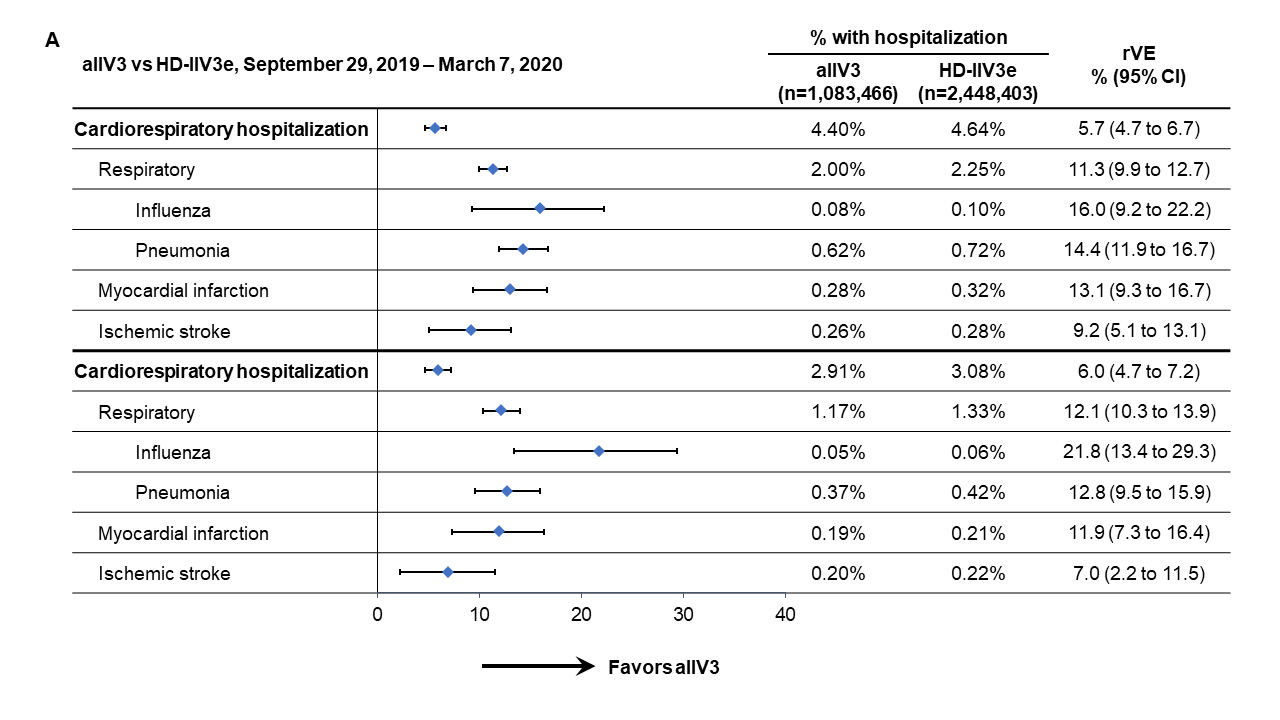


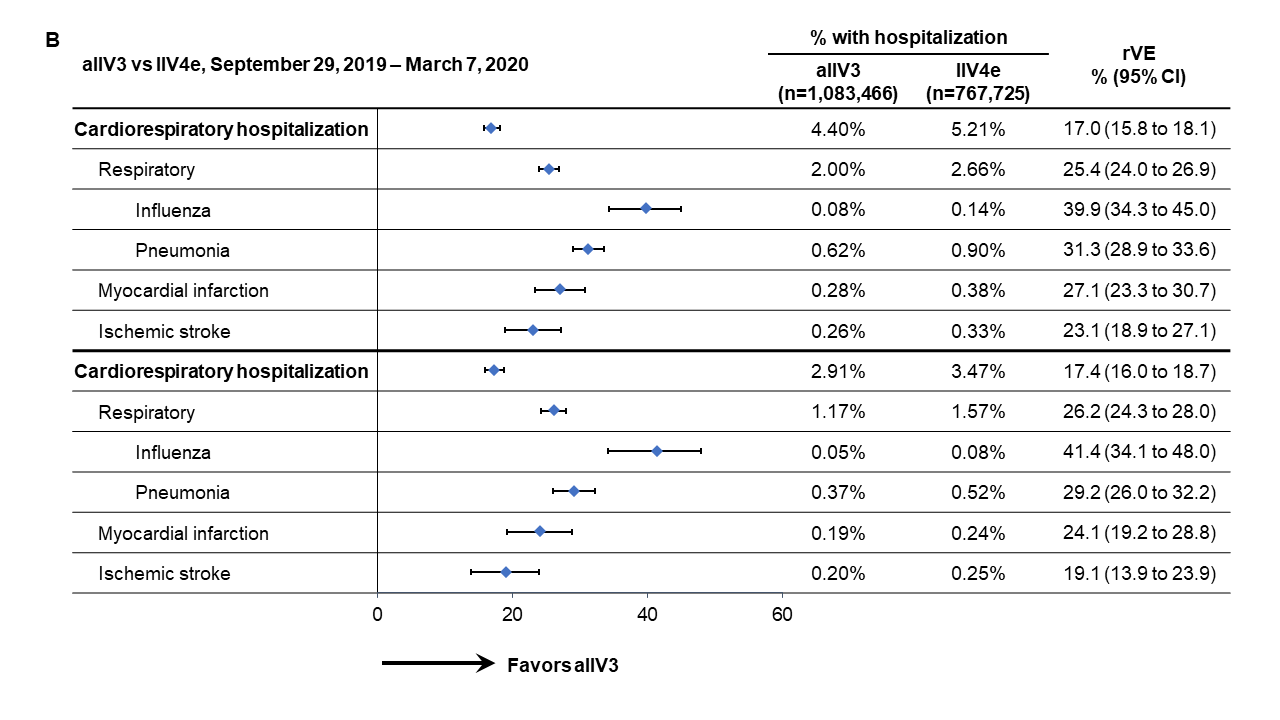


Table S7. Analyses of additional negative control outcomes between September 30, 2019, and March 7, 2020.

| **Hospitalization outcomes** | **aIIV3, n (%)** | **HD–IIV3e, n (%)** | **rVE, % (95% CI)** | **aIIV3, n (%)** | **IIV4e, n (%)** | **rVE, % (95% CI)** |
| --- | --- | --- | --- | --- | --- | --- |
| **Any position** |  |  |  |  |  |  |
| Appendicitis | 178 (0.016%) | 352 (0.014%) | –11.52 (–35.03 to 7.89) | 178 (0.016%) | 119 (0.016%) | –16.30 (–46.72 to 7.81) |
| Cataract | 897 (0.083%) | 1,832 (0.075%) | –1.32 (–10.30 to 6.94) | 897 (0.083%) | 695 (0.091%) | 3.32 (–7.46 to 13.02) |
| Medical device complications | 41 (0.004%) | 106 (0.004%) | — | 41 (0.004%) | 52 (0.007%) | — |
| Eyelid disorder | 164 (0.015%) | 275 (0.011%) | –19.48 (–46.57 to 2.60) | 164 (0.015%) | 92 (0.012%) | –36.24 (–78.68 to –3.89) |
| Hemorrhoids | 708 (0.065%) | 1,770 (0.072%) | 4.91 (–4.38 to 13.37) | 708 (0.065%) | 738 (0.096%) | 21.44 (12.37 to 29.57) |
| Herpes zoster | 138 (0.013%) | 407 (0.017%) | 16.76 (–2.29 to 32.27) | 138 (0.013%) | 132 (0.017%) | 18.18 (–5.70 to 36.67) |
| Lipoma | 67 (0.006%) | 178 (0.007%) | 0.99 (–33.06 to 26.33) | 67 (0.006%) | 67 (0.009%) | 18.99 (–15.80 to 43.33) |
| Nail disorders | 32 (0.003%) | 103 (0.004%) | 23.84 (–18.16 to 50.91) | 32 (0.003%) | 37 (0.005%) | 8.56 (–50.80 to 44.55) |
| **Admitting diagnosis** |  |  |  |  |  |  |
| Appendicitis | 150 (0.014%) | 293 (0.012%) | –12.27 (–38.44 to 8.96) | 150 (0.014%) | 93 (0.012%) | –28.30 (–65.77 to 0.71) |
| Cataract | 178 (0.016%) | 294 (0.012%) | –9.21 (–33.18 to 10.44) | 178 (0.016%) | 131 (0.017%) | 9.15 (–16.15 to 28.94) |
| Medical device complications | 3 (0.000%) | 4 (0.000%) | — | 3 (0.000%) | 0 (0.000%) | — |
| Eyelid disorder | 66 (0.006%) | 89 (0.004%) | –27.45 (–77.88 to 8.69) | 66 (0.006%) | 27 (0.004%) | –62.67 (–154.03 to –4.17) |
| Hemorrhoids | 94 (0.009%) | 235 (0.010%) | 8.34 (–18.82 to 29.30) | 94 (0.009%) | 109 (0.014%) | 21.29 (–5.45 to 41.25) |
| Herpes zoster | 45 (0.004%) | 107 (0.004%) | –10.05 (–58.52 to 23.61) | 45 (0.004%) | 41 (0.005%) | 16.11 (–33.30 to 47.24) |
| Lipoma | 17 (0.002%) | 61 (0.002%) | 38.47 (–6.31 to 64.39) | 17 (0.002%) | 17 (0.002%) | 20.34 (–58.81 to 60.04) |
| Nail disorders | 15 (0.001%) | 26 (0.001%) | –61.31 (–222.15 to 19.23) | 15 (0.001%) | 16 (0.002%) | –11.02 (–130.18 to 46.45) |

aIIV3, adjuvanted trivalent inactivated influenza vaccine (aIIV3); CI, confidence interval; high-dose, egg-based trivalent inactivated influenza vaccine; IIV4e, egg-based quadrivalent influenza vaccine; rVE, relative vaccine effectiveness.
